# In Patients Undergoing Cochlear Implantation, Psychological Burden Affects Tinnitus and the Overall Outcome of Auditory Rehabilitation

**DOI:** 10.3389/fnhum.2017.00226

**Published:** 2017-05-05

**Authors:** Petra Brüggemann, Agnieszka J. Szczepek, Katharina Klee, Stefan Gräbel, Birgit Mazurek, Heidi Olze

**Affiliations:** ^1^Tinnitus Center, Charité Universitätsmedizin BerlinBerlin, Germany; ^2^Department of ORL, Head and Neck Surgery, Charité Universitätsmedizin BerlinBerlin, Germany

**Keywords:** tinnitus, cochlear implants, psychological disorders, CIDI, psychological comorbidities

## Abstract

Cochlear implantation (CI) is increasingly being used in the auditory rehabilitation of deaf patients. Here, we investigated whether the auditory rehabilitation can be influenced by the psychological burden caused by mental conditions. Our sample included 47 patients who underwent implantation. All patients were monitored before and 6 months after CI. Auditory performance was assessed using the Oldenburg Inventory (OI) and Freiburg monosyllable (FB MS) speech discrimination test. The health-related quality of life was measured with Nijmegen Cochlear implantation Questionnaire (NCIQ) whereas tinnitus-related distress was measured with the German version of Tinnitus Questionnaire (TQ). We additionally assessed the general perceived quality of life, the perceived stress, coping abilities, anxiety levels and the depressive symptoms. Finally, a structured interview to detect mental conditions (CIDI) was performed before and after surgery. We found that CI led to an overall improvement in auditory performance as well as the anxiety and depression, quality of life, tinnitus distress and coping strategies. CIDI revealed that 81% of patients in our sample had affective, anxiety, and/or somatoform disorders before or after CI. The affective disorders included dysthymia and depression, while anxiety disorders included agoraphobias and unspecified phobias. We also diagnosed cases of somatoform pain disorders and unrecognizable figure somatoform disorders. We found a positive correlation between the auditory performance and the decrease of anxiety and depression, tinnitus-related distress and perceived stress. There was no association between the presence of a mental condition itself and the outcome of auditory rehabilitation. We conclude that the CI candidates exhibit high rates of psychological disorders, and there is a particularly strong association between somatoform disorders and tinnitus. The presence of mental disorders remained unaffected by CI but the degree of psychological burden decreased significantly post-CI. The implants benefitted patients in a number of psychosocial areas, improving the symptoms of depression and anxiety, tinnitus, and their quality of life and coping strategies. The prevalence of mental disorders in patients who are candidates for CI suggests the need for a comprehensive psychological and psychosomatic management of their treatment.

## Introduction

Cochlear implants (CI) make it possible for many people with hearing disorders to regain auditory perception and an acoustic understanding of language. The degree of improvement any particular patient experiences is influenced by complex, individual factors, leading to great variations in outcomes. A better grasp of the causes of this variation would improve our ability to predict the outcome for individual CI candidates.

One area of discussion has been the influence of concurrent psychological conditions on the neuronal plasticity of the auditory cortex. Alongside quantitative improvements in hearing, CI is known to have a positive effect on patients' quality of life (Olze et al., 2011, 2012b, 2016; Blasco and Redleaf, 2014; Ramos-Macias et al., 2016). Other influential factors include the progression of deafness, whether and how effectively hearing aids are used in rehabilitation, and motivational and psychological factors among patients—which can only be determined through a quantitative and qualitative measurement of their psychological burden.

In our earlier study of 43 patients with unilateral deafness who had undergone the cochlear implantation, we showed that tinnitus and concurrent psychological conditions had a significant influence on their quality of life (Olze et al., 2011). This study took into account the need for a qualified assessment of the patients' psychological conditions prior to and following implantation. The following year, we demonstrated that the CI patients with a high level of tinnitus-related distress had low quality of life, experienced more stress and more difficulties in coping with their situation (Olze et al., 2012a,b). From 2,251 language tests carried out on post-lingually deafened adults, Blamey et al. concluded that the level of successful postoperative hearing was negatively correlated with the length of the period of deafness prior to Cl (Blamey et al., 2013). Lin et al. observed a 60% improvement of speech recognition among patients over 60 years of age when assessed 1 year post-implantation. Beyond 60, each additional year was accompanied by a drop in the level of speech understanding of 1.3% (Lin et al., 2012). Several of these studies also employed standardized interviews such as the Composite Diagnostic Interview (CIDI), which revealed a range of psychological disturbances.

Van der Werf et al. described an association between psychological disturbances and limitations in hearing in a study of 3,021 youths and adults ranging from 14 to 24 years of age; the subjects were interviewed at regular intervals over a period of 10 years (van der Werf et al., 2011). The younger members of this group (aged 14–17) exhibited a higher incidence of psychological disturbances than those in the 18–24 age group. Their conclusion was that younger patients were going through a more sensitive period of development and as a result, their deafness had a greater impact on their psychological state and social interactions.

Mance and Edwards showed that a young Cl patient's psychological well-being correlated positively with the degree to which they considered themselves similar to people of their own age with normal hearing (Mance and Edwards, 2012). Fellinger et al. (2005) indicated that deaf patients are more commonly affected by somatic disturbances, fear and stress, than those with normal hearing. The deaf subjects had more social contact with other hearing-impaired people than with the unimpaired. The same group of hearing-impaired individuals generally had poorer social relationships than their deaf counterparts (Fellinger et al., 2007). All the scales that were employed yielded worse scores; in contrast to the truly deaf study participants, they were not simply members of a peer group with similar problems. This indicated that their quality of life depends on the level of contentment and success they feel within a system in which hearing plays an important role. The study concludes that the hearing-impaired are generally more isolated than those who are completely deaf, and that Cl can represent a successful form of assistance for this group.

In the present study, we aim to determine the occurrence of psychosocial burden, including the manifestation of psychological diagnoses, using questionnaires and a standardized interview in a subset of patients scheduled to undergo cochlear implantation. We formulated the following hypotheses:

(H1) that patients undergoing CI would suffer from a higher psychological burden than the general population;
(H2) that psychological burden is significantly reduced by the provision of a Cl;
(H3) that a lower-level of post-operative speech recognition improvement is associated with a higher level of psychological burden;
(H4) that certain dimensions of psychosocial limitations decrease the benefits of Cl.

## Methods

### Data collection

Between October 2010 and January 2012, within the framework of the Cochlear Implant Program of the Charité University Hospital in Berlin, 52 post-lingually, bilaterally deafened adults were interviewed prior to CI; 47 subjects were consecutively included in the study (Table 1), approved by a local Ethics Committee. All investigations were conducted according to the principles expressed in the Declaration of Helsinki. All patients gave their informed written consent.

**Table 1:** **Descriptive statistics**.

**Total number of patients**	**47**	
Gender	Women	Men
	30	17
Mean age (years)	56.09	63.08
Mean duration of deafness^*^ on the implanted side (in years)	15.4 (*SD* = 18.8)	
Side of implantation	21 left and 26 right	
Mean use of CI per day (in hours)	13.1 (*SD* = 3.7)	

* *According to subjective patient's reports*.

The mean value of FB MS on the implanted ear was for all 47 patients 3.08; *SD* = 8.8. On the contralateral ear, 36 patients were hard of hearing with a mean value of FB MS 16.1; *SD* = 24.0, whereas the remaining 11 patients had a mean value of FB MS 77.3; *SD* = 16.9.

The following procedures were applied to all patients prior to implantation and then half a year after they had received the Cl. The CIDI was carried out on a laptop computer. As questionnaires on paper, patients were administered the Oldenburg Inventory (OI), the Nijmegen Questionnaire (NCIQ), and the general perceived quality of life questionnaire (SF36). The following additional questionnaires were completed using a pocket computer: General- Anxiety Disorder (GAD), General Depression Scale (ADS), the COPE Inventory, the tinnitus questionnaire (TQ), and the Perceived Stress Questionnaire. Audiometric measurements were performed in the Audiometry Unit of the ORL, Head and Neck Surgery Department.

The consent of the Ethics Commission was obtained prior to the study. All patients consented to their participation in the interviews and questionnaires.

### Composition of the patient sample

The cohort for this study was comprised of 30 women and 17 men who averaged 58.62 years of age and a period of deafness lasting 15.4 years averaged over the group. The cause of deafness varied between the patients: unknown cause of deafness was stated by 19 patients; recurrent otitis media in the childhood was reported by 6 patients; sudden sensorineural hearing loss was reported by 4 patients; noise was reported by 3 patients; meningitis in 2 patients. Meniére's disease, otosclerosis, hypoxia at birth, cholesteatoma, autoimmune diseases, familial hearing loss, and childhood violence were given once as a reason for deafness. Six patients did not answer this question. The period of deafness was determined based on the statement about the time when a patient felt that wearing a hearing aid stopped being of significant benefit. Most of the patients had secondary school education and had completed professional training, they were in a long-term relationship and, as expected given the high average age, were retired. The causes of their hearing loss correspond to the types and distribution generally found in data from literature on this topic.

### Composite international diagnostic interview

The CIDI was developed in the context of epidemiological studies by the World Health Organization (WHO) (Robins et al., 1988). The interview is used in both a Short Form and the DIA- X/M- CIDI Münchner Composite International Diagnostic Interview (Wittchen, 1994). For this project the latter was used, specifically version 1.2 from 13.08.1999. Its goal is to assess psychological and behavioral disturbances according to ICD-10 and DSM-IV, and its contents are divided into several sections (A through X) and three supplementary sections, listed in Table 2. Not all of the sections were employed in this study because to do so would have represented an undue burden on patients' time. Single interviews lasted between 30 and 90 min. Questions covered time frames ranging from the previous 2 weeks to 12 months and extended to the patient's entire lifespan. The interview can be conducted by anyone; no prior clinical or diagnostic expertise is required. The Interrater Reliability of the CIDI lies at 0.81–1.0. The reliability of retesting for single diagnoses of somatic conditions lies between 0.49 and 0.67; for affective disturbances the value lies at 0.77, for single diagnoses between 0.45 and 0.69, and for anxiety diagnoses between 0.57 and 0.72.

**Table 2:** **Sections of CIDI**.

**Section**	**Description**	**Included in this study**	**ICD10 code**
A	General questions	X	
B	Disorders resulting from the use of tobacco		F17
C	Somatoform disorders	X	F45
D	Phobic disorders	X	F40- F41
E	Depression (affective disorders F30- F39)	X	F32- F33
F	Manic episode (affective disorders F30)		F30
G	Schizophrenia and schizoaffective disorders		F20- F29
H	Eating disorders	X	F50
I	Psychological and behavioral disorders caused by alcohol	X	F10
K	Obsessive-compulsive disorder	X	F42
L	Disorders resulting from the use of medications other substance abuse	X	F11- F19
M	Organic disorders (including symptomatic mental disorders)		F0- F09
N	Post-traumatic stress disorder	X	F43
ML	Munich task list		
Q	Final questions		
P	Interview observations		
X	Interview judgment		
FR	Family genetics		
SQ	Personal and other questions		
RL	Restless-leg syndrome Questionnaire		

### Audiometric testing procedure: freiburg monosyllable test in quiet (FB MS)

The Freiburg monosyllable speech discrimination test (Hahlbrock, 1953) involves presenting patients with 2 × 20 monosyllabic words at a volume of 65 dB HL under quiet conditions. The patient's task is to repeat each word. The FB MS can be selected from 10 test lists; here lists 9 and 10 were used. Each correctly repeated word counts for 5% of the comprehension score. The test was carried out on each ear separately prior to the CI procedure (noted as the “ear to be implanted,” and “opposite ear”). Following Cl implantation, measurements were carried out using the same lists of words. As on the ear to be implanted, the average speech comprehension prior to CI was only about 3%, the learning bias was excluded. On the non-CI ear, the average speech comprehension was 16.1% for 36 patients and 77.3% for the remaining 11 patients. Any patients who wore a hearing aid in the “opposite ear” had to remove it prior to the test. The words were presented *via* speakers and the non-CI ears were plugged.

### Testing the quality of hearing by questionnaire

#### Oldenburg inventory

The OI (Kollmeier and Holube, 1991) originally consisted of 21 items with 5 subscales. The current study used the shortened version of the questionnaire with 12 questions and 3 scales: Listening in a quiet setting (Questions 1, 3, 5, 7), listening with noise interference (4, 6, 8, 11, 12) and directional listening (2, 9, 10). The 12 closed questions about everyday situations were marked with points from 1 to 5. The range for each item of the 3 subscales of the Ol was chosen from 1 to 5 which adds up to a total range from 12 to 60 for the 12 questions. Average scores for each subscale was used as well as the overall result ranging from 1 to 5. The higher the score, the better the subjective hearing.

#### Nijmegen cochlear implantation questionnaire

The purpose of the NCIQ (Hinderink et al., 2000) is to establish the quality of a patient's life prior to being outfitted with a CI and again afterwards. It is based on 6 scales and consists of 60 items, listed in Table 3. The NCIQ scores of the subscales as well as the total score are normalized to percentages. When used in studies of patients with CI, questions about contentment and the time the device has been worn are added to the NCIQ.

**Table 3:** **Domains of the Nijmegen Cochlear Implant Questionnaire (NCIQ)**.

**Domains**	**Content**	
Basic sound perception	Background sounds	NCIQ1
Advanced sound perception	Ability to communicate	NCIQ2
Speech production	Voice monitoring	NCIQ3
Self-esteem (psychological domain	Communicative skills	NCIQ4
activity	Personal and professional	NCIQ5
Social interactions	Communication with family, friends, peer groups	NCIQ6
NCIQ total	Score for health-related quality of life	NCIQ total

### Psychometric questionnaire to establish psychological burden

#### SF36: questions regarding general health

SF36 (Piehlmeier et al., 1996) is a German translation of the American SF36 Health Survey (Jenkinson et al., 1993) and consists of 8 scales. The questions relate to a patient's state of health and query an individual's overall perception of his or her state of health for the period of 1 week. Estimations of psychological and bodily health are scored separately and appear as individual values in the results provided here.

#### Tinnitus questionnaire

The German version of TQ (Goebel and Hiller, 1994) aims to establish the degree of severity of a patient's tinnitus and consists of 52 items distributed in 6 scales. Scores can range from 0 to 84 and are evaluated as follows: a 1st degree burden falls within the range 0–30; 2nd degree, 31–46; 3rd degree, 47–59; and 4th degree from 60 to 84. Scores under 47 are considered “compensated” tinnitus and all higher scores are termed “decompensated.” The compensatory type represents a form of tinnitus that is chronic and yet does not impose grave restrictions on a patient's life.

#### Perceived stress questionnaire

The German version of PSQ (Fliege et al., 2005) delivers a patient's subjective perception of stress factors. It comprises 30 items grouped into 7 scales designated as the following: Harassment, Overload, Fatigue/Irritability, Lack of Joy, Worries, and Tension. This study relies on the shortened version of the scale. Every scale can have values from 0 to 1. A score above 0.45 is considered to represent a moderate level of stress; anything above 0.6 is termed high stress.

#### COPE inventory

COPE (Carver et al., 1989) is intended to investigate a patient's personal resources and modes of coping with stress. The shortened German Brief-COPE version used in this study contains 4 scales: avoidance, active problem solving, positive thinking, support-seeking behavior (compare with Knoll et al., 2005), whereas the answer scores may range between 0 and 30.

#### General anxiety disorder 7 questionnaire

GAD-7 (Spitzer et al., 2006) investigates the frequency and degree to which a patient has experienced fear within the 2-week period preceding the questionnaire. Seven items are measured and scored based on patients' responses, using the following scale: 0 = not at all; 1 = on some days; 2 = more often than every other day; 3 = almost every day. A sum of the scores provides a value for estimating the degree of the burden presented by fear (small, mild, medium or strong). The answer scores range between 0 and 21.

#### General depression scale

The ADS (Mohiyeddini et al., 2002) aims to determine the presence, degree, and length of depression a person has experienced within week immediately prior to the questionnaire. 20 items are covered. The points are added to achieve a total score that ranges from 0 to 60. A score over 23 is considered to describe a condition of serious depression. When the ADS is administered to members of the general public, the average score is 14.30 (SD 9.7). The median score achieved by patients diagnosed with depression is 36.70 (SD 8.4), compared to those who experience states of fear or social phobias, who have on the average a median score of 36.60 (SD 8.71).

### Statistical procedure

Forty-seven patients were included in this study. The statistical evaluation was carried out with the software program SPSS (IBM Corp. Released 2015. IBM SPSS Statistics for Windows, Version 23.0.0.2, Armonk, NY: IBM Corp.). Frequency tables, mean values and standard deviations were calculated descriptively (Table 2). The questionnaires and CIDI were evaluated using a nonparametric ANOVA (Friedman Test of relevant samples), once the requirements for the *t*-test of samples were achieved, to test the significance of differences before and following implantation (H2). To clarify the type of association and the influence of various parameters on progressive hearing ability, regression models were calculated, in which the performance in pre- and post-language audiograms were defined as dependent variables.

## Results

### Hypothesis 1: that patients undergoing CI would suffer from a higher psychological burden than the general population (as measured with CIDI)

**Figure 1:**
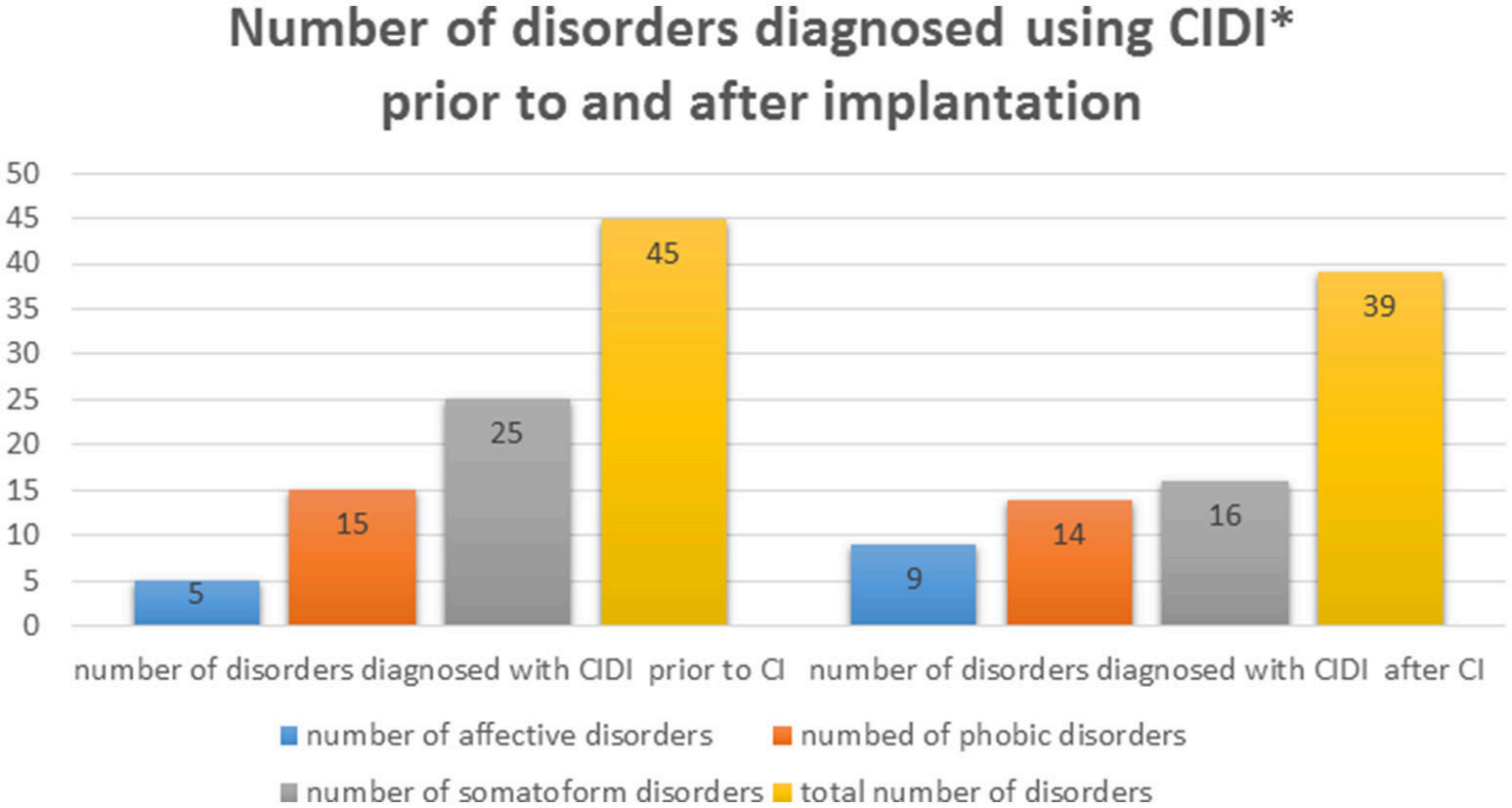
**Absolute number of psychological disorders diagnosed in our sample of implanted patients before and after CI, as determined through CIDI**. ^*^One subject can be diagnosed with more than one disorder.

Eighty one percent of patients had psychological disturbances before and/or after CI, which is in contrast to the incidence of psychological disturbances in general German population (32.1% in people between 18 and 65 years). In 13% of the cases, patients were diagnosed with a disturbance only prior to CI but not afterwards, while 11% received this diagnosis only afterwards (Figure 1).

**Figure 2:**
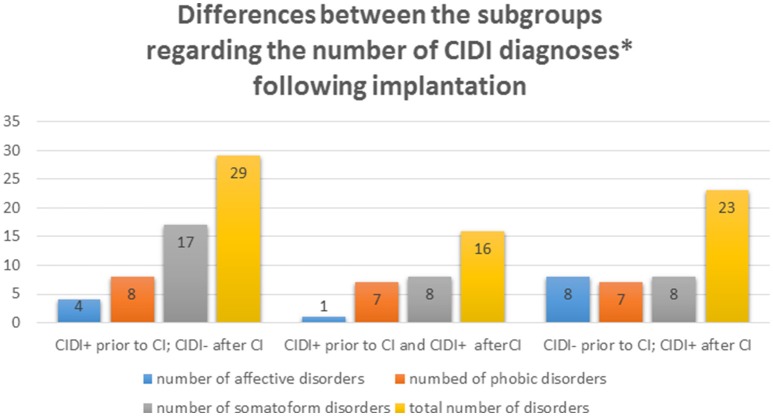
**Differences between the numbers of diagnoses occurring in three subgroups of patients**. First subgroup represents positive diagnoses of psychological disorders prior to CI and their disappearance after CI. Second subgroup represents positive diagnoses prior to and after CI. The third subgroup represents presence of diagnoses following CI. ^*^One subject can be diagnosed with more than one disorder.

Prior to implantation, 11% were diagnosed with affective disturbances, 32% suffered from anxiety and 53% from psychosomatic disturbances. Following CI, 11% of the patients exhibited one or more affective disturbances, 30% had anxiety and 34% a psychosomatic illness (Figure 2). One patient was diagnosed with obsessive-compulsive disorder before and after CI. Another exhibited a post-traumatic stress disorder prior to CI that was no longer apparent post-implantation based on the CIDI diagnosis.

Hypothesis 1 is regarded as confirmed based on the overall higher psychological comorbidity of relevant diagnoses in the patient group for the areas of anxiety and psychosomatic disturbances, when compared to the general population. These values change only marginally after cochlear implantation. A moderate decrease of psychosomatic disturbances is observed, although it does not reach significance.

### Hypothesis 2: that the psychological burden is significantly reduced by the provision of a Cl

Here we do not present results related to social data and age, sex, length of wearing the device, contentment, or length of deafness that fail to reach significance, with the exception of the stress level (PSQ). Descriptive results for all of the significant changes in the variables that were evaluated are summarized in Table 4.

**Table 4:** **Test scores prior to and following CI (means, significance levels)**.

	***N***	**Range**	**mean before CI**	**Standard deviation**	**mean after CI**	**Standard deviation**	***Z***	***p* (<0.05)**
FB MS	47	0–87.5	3.08	8.81	28.27	28.72	4.394	0.00001
OI quiet setting	45	1.0–4.6	2.56	0.77	3.34	0.85	4.114	0.00004
OI noise interference	45	1.0–4.4	1.93	0.65	2.74	0.80	4.939	0.000001
OI directional listening	45	1.0–4.5	2.10	0.73	2.78	0.95	3.749	0.0002
OI total	45	1.0–4.6	2.22	0.62	3.07	0.70	5.246	0.000000
NCIQ 1	46	0–92.5	45.32	20.95	61.23	17.44	3.74	0.0002
NCIQ 2	47	12.5–78	45.48	16.62	58.26	17.14	4.19	0.00003
NCIQ 3	47	25–82.5	64.02	18.11	71.51	15.86	2.912	0.004
NCIQ 4	46	20–82.5	43.79	16.94	51.54	18.12	2.68	0.007
NCIQ 5	47	0–94.2	41.56	18.29	53.57	18.27	4.342	0.000014
NCIQ 6	46	10–100	44.42	19.37	57.58	22.46	3.960	0.00008
NCIQ total	46	11.4–94	47.28	13.43	59.00	14.94	4.518	0.000006
SF36 physical health	46	21.5–64	52.07	7.48	45.21	10.51	4.357	0.00001
SF36 psychological health	46	15.5–62	42.91	10.72	48.33	9.87	3.414	0.0006
TQ emotional distress	47	0–20	7.19	6.11	5.15	5.77	2.414	0.016
TQ cognitive distress	47	0–14	5.19	4.86	3.808	4.84	3.472	0.0005
TQ intrusiveness	47	0–15	6.34	4.74	4.49	4.53	2.738	0.06
TQ auditory perceptual difficulties	47	0–14	5.70	4.91	3.85	4.26	2.451	0.01
TQ sleep disturbance	47	0–6	1.70	2.21	0.97	1.79	2.259	0.02
TQ somatic complaints	47	0–5	1.00	1.47	0.62	1.21	1.980	0.04
TQ total	47	0–72	27.66	22.01	18.70	19.34	3.001	0.002
PSQ worries	47	0–0.67	0.31	0.18	0.28	0.18	1.87	0.06
PSQ tension	47	0–0.93	0.36	0.22	0.34	0.23	0.726	0.468
PSQ joy	47	0.07–1	0.62	0.23	0.58	0.27	1.400	0.161
PSQ demands	47	0–0.8	0.28	0.18	0.27	0.20	0.570	0.569
PSQ total	47	0–0.69	0.34	0.17	0.33	0.18	0.638	0.524
COPE avoidance	43	7–22	11.74	2.74	10.21	2.97	2.346	0.02
COPE seeking support	43	7–21	13.98	3.4	12.74	3.62	2.718	0.007
COPE positive thinking	43	6–22	13.85	3.04	13.40	3.82	0.705	0.481
COPE active problem solving	43	4–16	10.53	2.59	9.33	2.80	2.056	0.04
Depression (ADSL)	47	5–41	17.93	10.84	14.40	9.24	2.336	0.02
GAD7	47	0–21	5.64	4.69	3.81	4.01	3.057	0.002

All of the results related to hearing capacity show a highly significant degree of improvement. They include improvements of the auditory abilities measured by objective (audiometry in quiet and noise and directional hearing) and subjective evaluations (NCIQ, Ol).

All subscores of the Tinnitus Questionnaire and thus the combined total score indicating tinnitus-related distress (TQ) showed significant improvement. The average TQ score decreased significantly from 27.66 prior to CI to 18.70 post-CI (*p* > 0.01; for the subscales see Table 4). No significant changes were seen in overall stress perception (PSQ), or in its subscores. The coping mechanisms in COPE changed in the direction of a lower utilization of health care resources. Anxiety (GAD7) and depression (ADS) revealed significant statistical improvement.

All of the self-evaluated and audiometrically measured parameters exhibited significant improvements following CI. Accordingly, we regard hypothesis 2 as confirmed.

### Hypothesis 3: that a lower-level of post-operative speech recognition improvement is associated with a higher level of psychological burden

The analysis of how the psychological disturbances affect the measured parameters, revealed that the patients *without* psychosomatic, anxious or depressive illnesses had significantly better scores in almost all subcategories of the questionnaires before and after cochlear implantations. Across the board in these groups, there was significant improvement in speech comprehension.

Independently of the presence of psychosomatic conditions, changes in the TQ scores indicated significant effect of CI on the emotional burden, hearing problems, sleep disorders or psychosomatic complaints related to tinnitus. Their experience of stress underwent no significant change, with the exception of a significant drop in their level of concern (PSQ). No significant changes were noticed in any subscore of the COPE. Patients without an affective disturbance did not experience a significant change as measured either PSQ or COPE scores between their preoperative and postoperative measurements. This was also the case for patients with anxiety.

### Group with psychological illness prior to CI but none afterwards

Patients who exhibited a psychological disturbance prior to CI but none after implantation could be characterized as follows. Those who were relieved of a psychosomatic illness had better results in FB MS, on all subscores of the OI, basic sound perception (NCIQ1), advanced sound perception (NCIQ2), activity (NCIQ5), emotional and cognitive burden through tinnitus with a significantly poorer performance in SF36 KSS. Patients with anxiety whose condition vanished after CI achieved better scores in directional hearing (OI), active behavior (NCIQ5), the establishment of social interactions (NCIQ6) and active stress management (COPEac).

### Group with psychological illness before and after CI (including new conditions)

Patients whose psychological illness remained after CI, or who exhibited a new condition following the procedure, exhibited notably fewer significant changes overall in the parameters that were measured. The psychosomatic patients improved only in the parameters of hearing accompanied by distracting noise (OI), the establishment of social interactions (NCIQ6), psychological well-being (SF36 PSS), avoidance behavior (COPE ec) and assistance-seeking (COPE sc). The group with anxiety before and after CI showed significant improvement only in hearing accompanied by distracting noise (OI) and in basic sound perception (NCIQ1). This group had, however, significantly poorer results in measurements of physical well-being (SF 36 KSS) and experience of joy (PSQ joy).

The group in which new psychosomatic disturbances appeared had significantly better scores in the subareas: hearing accompanied by distracting noise (OI), speech production (NCIQ3), self-esteem (NCIQ4), activity (NCIQ5), social interactions (NCIQ6), and assistance-seeking behavior (COPEsc), while at the same time achieving significantly lower results for total bodily health (SF36 KSS).

Patients who exhibited new affective illnesses after CI had better results in the Freiburg monosyllable test, hearing against a quiet background (OI), advanced sound perception (NCIQ2), active behavior (NCIQ5) and social interactions (NCIQ6). They had significantly poorer results in total physical scores in SF36.

Patients diagnosed with anxiety disorders after but not prior to CI, showed significant improvement only in directional hearing (OI), social interactions (NCIQ6) and psychosomatic complaints through tinnitus (TQ SO).

### Associations between psychological burden and effects of CI in the group as a whole

Overall our work shows that the high-grade anxiety or depression present before (and after) CI lead to poorer estimates of acoustic perception (OI and NCIQ) than in patients who had low-grade of anxiety or depression (Table 5). Subjective estimates of a good quality of life (SF36) correlate positively with estimates regarding auditory perception. The benefits of speech recognition (FB MS) demonstrate a negative correlation with the length of deafness. The physical aspects of quality of life are initially reported at a lower level than these benefits, but over time this trend seems to reverse itself. Overall, older patients don't seem to profit as quickly as younger patients with regard to the perception of the quality of hearing, and they estimate their physical quality of life at lower levels before the implantation; the same is true of their estimates of contentment.

**Table 5:** **Correlations for pre- and post-implantation results**.

	***N***	**Pearson's correlation**	**Significance (<0.05)**
DELTA MS (changes language hearing) and SF 36 psychological	45	−0.394	0.021
DELTA MS (changes language hearing) and duration deafness	45	−0.308	0.059
age and NCIQ (acoustic aspects quality of life) pre	46	−0.321	0.051
age and NCIQ (acoustic aspects quality of life) post	46	−0.321	0.051
age und satisfaction with CI	45	−0.488	0.005
age und SF 36 (bodily health) pre	46	−0.671	0.013
time after implantation and SF 36 PSS (psychological health) pre	45	0.275	0.030
NCIQ total (acoustic QOL) pre and satisfaction	46	0.468	0.007
NCIQ total (acoustic QOL) pre und OL (hearing quality) pre CI	46	0.441	0.011
NCIQ total (acoustic QOL) pre und OL(hearing quality) post CI	46	0.565	0.001
NCIQ total (acoustic QOL) pre and GAD pre CI	46	−0.434	0.012
NCIQ total (acoustic QOL) pre and ADS pre CI	46	−0.357	0.034
NCIQ total (acoustic QOL) post and satisfaction with CI	46	0.453	0.009
NCIQ total (acoustic QOL) post and duration deafness r	46	0.429	0.013
NCIQ total (acoustic QOL) post und OL (hearing quality) pre CI	46	0.549	0.002
NCIQ total (acoustic QOL) post und OL (hearing quality) post CI	46	0.440	0.011
NCIQ total (acoustic QOL) post and GAD (anxiety) pre CI	46	−0.368	0.030
NCIQ total (acoustic QOL) post and ADS (depression) post CI	46	−0.317	0.053
NCIQ total (acoustic QOL) post and SF 36 KSS (somatic aspects quality of life) post	46	0.388	0.013
NCIQ total (acoustic QOL) post and SF 36 PSS (psychological aspects quality of life) post	46	0.521	0.002
OI (hearing quality) pre CI and duration of deafness	46	0.406	0.018
OI (hearing quality) pre CI and satisfaction	46	0.385	0.024
OI (hearing quality) post CI and duration of deafness	46	0.390	0.022
OI (hearing quality) post CI and satisfaction	46	0.399	0.019
OI (hearing quality) post CI and ADS (depression) post CI	46	−0.327	0.048
OI (hearing quality) post CI and TF pre CI	46	0.418	0.015
OI (hearing quality) post CI and time after implantation	46	0.312	0.057
TQ (tinnitus distress) post and duration of deafness	47	0.316	0.054
TQ (tinnitus distress) post and satisfaction	47	−0.313	0.056
TQ (tinnitus distress) post and GAD (anxiety) post CI	47	0.638	0.000
TQ (tinnitus distress) post and ADS (depression) pre CI	47	0.393	0.021
TQ(tinnitus distress) post and ADS (depression) post CI	47	0.406	0.018
GAD (anxiety) pre CI and ADS (depression) pre CI	47	0.650	0.000
GAD (anxiety) pre CI and ADS (depression) post CI	47	0.357	0.034
GAD (anxiety) pre CI and SF 36 KSS (bodily health)	47	−0.364	0.026
GAD (anxiety) pre CI and SF 36 PSS (psychological health)	47	−0.426	0.010
GAD (anxiety) pre CI and stress pre CI	47	0.800	0.000
GAD (anxiety) pre CI and stress post CI	47	0.678	0.000
GAD (anxiety) post CI and duration deafness	47	0.444	0.010
GAD (anxiety) post CI and TQ(tinnitus distress) post	47	0.638	0.000
GAD (anxiety) post CI and stress post CI	47	0.535	0.002
GAD (anxiety) post CI and depression post CI	47	0.589	0.001
ADS (depression) pre CI and stress pre CI	47	0.787	0.000
ADS (depression) pre CI and stress post CI	47	0.744	0.000
ADS (depression) pre CI and SF 36 PSS (psychological aspects quality of life)	47	−0.620	0.002
ADS (depression) post CI and stress pre CI	47	10.53	0.04
ADS (depression) post CI and stress post CI	47	17.93	0.02
ADS (depression) post CI and SF 36 PSS (psychological health)	47	5.64	0.002

*Shown are only significant results, with no correlations between tests*.

We were able to partially confirm hypothesis 3: that the level of psychological burden is accompanied by a decrease in the profit perceived/obtained from CI. This is particularly true in cases of symptoms of anxiety and depression, which potentially influence the target improvement in hearing capacity through their effects on quality of life.

### Hypothesis 4: that certain dimensions of psychosocial limitations decrease the benefits of Cl

To determine what influence CIDI-diagnosed conditions have on the effects of CI in terms of audiometrically determined speech recognition, we calculated a linear regression. As predictors, the presence of symptoms of affective, psychosomatic and anxiety disturbances were considered; the time of wearing a hearing aid and level of contentment were used as controls. The overall regression model was determined not to be significant (*F* = 1.175, *p* < 0.344).

Post-operative statistics reveal a tendency toward effects related to psychosomatic and anxiety disturbances. Patients who suffered from these types of conditions before and after the implantation exhibited less of a gain in audiometrically measured speech recognition. This effect was already evident in correlations related to the questionnaires (see Table 6), but only shows a tendency toward significance. Overall, in explaining the significant improvements in all areas of auditory perception and particularly language perception of the group of patients who underwent cochlear implantation, the presence of a psychological disturbance does not appear to be relevant.

**Table 6:** **Regression model: coefficients of predictors for speech perception after CI**.

**Model**		**Unstandardized coefficients**		**Standardized Coefficients**		***t***	**Significance**
		***B***	**Std. Error**	**Beta**			
1	(Constant)	28.834	24.786			1.163	0.254
	Satisfaction	0.002	0.203	0.002		0.011	0.991
	Compliance (duration of CI per day)	1.116	1.559	0.123		0.716	0.479
	Affective disorder before but not after CI	20.601	19.737	0.197		1.044	0.305
	No affective disorder before but after CI	21.656	15.895	0.247		1.362	0.183
	Phobic disorder before but not after CI	−25.313	15.896	−0.373		−1.592	0.121
	No phobic disorder before but after CI	−25.820	14.500	−0.313		−1.781	0.085
	Phobic disorder before and after CI	28.958	24.032	0.306		1.205	0.237
	Somatoform Disorder before but not after CI	−14.527	12.848	−0.230		−1.131	0.267
	No somatoform disorder before but after CI	−31.122	18.030	−0.378		−1.726	0.094
	Somatoform disorder before and after CI	−28.909	18.616	−0.330		−1.553	0.131

*Dependent Variable: Delta in speech perception (Freiburg monosyllable speech perception test)*.

Hypothesis 4 regarding the influence of diagnoses related to psychological factors cannot be confirmed, but psychosomatic effects and anxiety before the surgery seem to have an influence on the negative effect hearing specifics after CI.

## Discussion

### Psychological diagnosis and hearing deficiencies

In Germany, approximately 32.1% of the population aged 18–65 is affected by psychological disturbances. Figures from the Federal German Retirement Insurance system state that in the year 2014, 43% of the population reported having been affected by such problems at least once in their lives; the results are a reduction in performance and a retreat from social life. These disturbances are the most common reason for work-related disabilities in Germany, which is a key motivation to develop effective diagnoses and treatment. In our survey of 47 deaf patients, 31 had a psychological diagnosis before the implantation, a figure which is significantly higher than the average in the general population. Following the intervention, 25 patients (over half of the measured group) had such a diagnosis, which is still much higher than the standard reference. We regard this as relevant despite methodological criticisms which have been raised concerning the form of the interview that was used.

It is impossible to say how reliable and valid the results of the diagnoses captured with CIDI are in relation to clinical diagnoses. Becker et al. regard the use of CIDI as insufficient; in a study of the degree of consistency between clinical diagnoses and the use of CIDIs, they found Kappa values between 0.0 and 0.33 for all diagnoses (Becker et al., 2006). Such unsatisfactory values for the validity and reliability of the CIDI are partially confirmed by the literature (Semler et al., 1987). The important coping disturbances in deaf patients are not covered in these estimates (Hund et al., 2014). Kessler et al. reported good rates of correspondence between clinical diagnoses and standard interviews (Kessler et al., 2009). Knappe et al. consider the use of standardized interviews such as CIDI the “gold standard” (Knappe et al., 2008). In spite of these methodological issues, in agreement with data, we believe that the higher level of psychological diagnoses in our sample is an accurate representation of the patients' situation. In this context, we do not consider the burden of biopsychological and social factors related to deafness and potential side effects of the treatment to be the only causes of the higher level of psychological diagnoses. Some of the problems may be neurological; animal experiments have shown that there are direct connections between the acoustic cortex and the limbic system, and these also may play a role (Kraus and Canlon, 2012).

Our results are in agreement with data from the literature. In the current patient cohort, CIDI diagnoses could be broken down into three major groups: affective, anxiety-related, and psychosomatic disturbances. The diagnoses were commonly the following: for affective disturbance, dysthymia (coping disturbances); in anxiety: agoraphobia and other phobias of an indistinct nature; psychosomatic disturbances: pain and undefined psychosomatic conditions (possibly including tinnitus). Using CIDIs, we diagnosed frequent affective, psychosomatic and anxiety disorders in patients with chronic tinnitus (Zirke et al., 2013). This is in agreement with high prevalence of psychological disorders diagnosed in patients with other chronic somatic diseases, such as cardiovascular diseases (Baumeister and Harter, 2011) or kidney dysfunction (McClellan et al., 2010).

### Improvement of burden following CI

The loss of binaural hearing is associated with a decreased speech comprehension in noise and problems with sound localization, even when the unaffected ear is healthy. Additionally, in CI patients diagnosed with unilateral hearing loss and tinnitus, they showed improvement in speech understanding in noise following implantation (Blasco and Redleaf, 2014). Our study revealed a significant improvement in speech discrimination following CI (measured with FB MS, which is a standard component of measuring the speech recognition). Subjective hearing abilities increased in all subscales (OI), as was the case for all the subdomains of the NCIQ. Parallel use of audiometric and psychometric evaluations leads to consistent measurement of the CI effects.

Today, the improvement of speech recognition that is achieved through CI is presumed to be linked at least in part to the neuronal plasticity of the cortex. The ultrastructural organization of each component of the auditory system (periphery, cochlear nucleus and auditory midbrain and the auditory cortex) undergoes degradation if it is not stimulated (Baizer et al., 2015). Anatomical and physiological changes have all been shown to be at least partly reversible with renewed stimulation of the cochlear nerve, even considering that certain periods are more sensitive than others. This reorganization occurs in many areas: those that are directly connected to the cochlear nerve, those anatomically linked to it, more distant regions, and in homologically related areas of the other brain hemisphere (Hotting and Roder, 2013). In a review, Kral also addressed the issue of sensitive periods and visual/auditory cross-modal reorganization in adults who become deaf post-lingually (Kral, 2007). In imaging studies, a hypometabolism of the auditory cortex was predictive of better speech perception following CI (Lee et al., 2007). Our present study deals with the connectivity between the auditory cortex (possibly undergoing plastic changes) with other cortical areas like limbic system or the cortical areas responsible for attention.

Here TQ, COP, PSQ, ADS, and GAD7 revealed significant improvements, also confirming the results of previous studies. All of the subscales of the TQ achieved better scores. Earlier we reported a 39.2% drop in the burden of tinnitus after 2 years, and none of the patients without tinnitus exhibited worsening in this area (Olze et al., 2012b, 2016).

The effects of tinnitus are also considered highly connected to basic principles of synaptic and cortical plasticity (Mazurek et al., 2010; Georgiewa et al., 2016). Synaptic plasticity is defined as the degree to which the strength of synaptic transmission changes through activity. The repeated stimulation of afferent processes can lead to changes through long-term potentiation (LTP), whereas decreases in stimulation can cause long-term depression (LTD). Damage to the central or peripheral auditory systems can lead to an imbalance between LTP and LTD, and consequently to changes in the activity of ion channels, receptors, and neuronal transmission. Cellular hyperactivity may induce hyperactivation of the central auditory pathways and change cortical plasticity in ways that lead to tinnitus. A restoration of acoustic stimulation can be achieved through cochlear implants, and in the best-case-scenario this may lead to a reorganization of synaptic plasticity in ways that are accompanied by a reduction in tinnitus, which we observed in our sample using the TQ.

The COPE Inventory revealed a decrease in the degree to which patients rely on coping strategies following CI. In other words, a significant drop in efforts required to master a situation was observed after CI. This finding is consistent with Kobosko et al. (2012), who found that 78 post-lingually deafened adult patients employed less active coping strategies and more avoidance following CI.

The values we obtained here for HRQoL (SF-36) showed significant drops in total scores for physical factors, whereas psychological scores rose. However, our overall results suggest general improvements in the quality of life, corroborating similar findings of Arnoldner et al., who also reported inconsistencies in SF 36 with regard to the quality of life in a 10-year study following cochlear implantations (Arnoldner et al., 2014).

Perceived stress (PSQ) revealed no significant changes following CI but the scores of depression (ADS) and anxiety (GAD7) demonstrated statistically significant improvements. We speculate that this could reflect stronger connectivity between the auditory and limbic systems (amygdala) than this between the auditory system and HPA axis. At both measurement time points, ADS scores lay higher than those for the general population (*M* = 14.30), but clearly below the cut-off of 23, which is considered the level at which a patient is considered manifestly depressive. The scores we obtained were 17.93 ± 10.84 (prior to CI) and 14.40 ± 9.24 (post-CI).

### The effects of psychological disturbances on the success of treatment

The significant improvements in auditory and psychosocial parameters that we measured were higher for patients without psychological disturbances than for those who had been diagnosed. The former group enjoyed greater benefits in terms of the quality of life, their perception of tinnitus and speech recognition. This does not, however, seem to be directly related to the presence of preexisting psychological diagnoses. We were unable to confirm hypothesis 4 using a regression model to account for changes in speech comprehension. We interpret the correlative data in Table 6 as potential evidence for an indirect link between the degree of psychological burden and the success of treatment. One factor to consider in this regard is that the neuronal plasticity of the auditory cortex permits an improvement of hearing after CI even in patients with high levels of burden. The degree of improvement is clearly lower, however, in patients suffering from depression or anxiety. This too, in our opinion, may be due to connectivity within the hearing networks of the brain, which are thought to directly link the auditory cortex with the amygdala (Kraus and Canlon, 2012). Existing emotional burdens could lead to defects in this network. Another issue that will require further reflection is whether affective disorders—or the limitations in learning and memory that accompany them—have a direct impact on the mechanisms underlying neuronal plasticity.

Clinicians have long recognized that patients burdened by both a physical illness and a psychological disturbance have a lower life expectancy, poorer compliance, and poorer quality of life than those who exhibit only physical symptoms. The identification of accompanying psychological disturbances is a basic requirement in establishing a patient's right to rehabilitative care and is also crucial in predicting the likely effect of therapies on individuals (Blasco and Redleaf, 2014). Past observations by our group also indicate that patients with chronic tinnitus achieved significantly better scores not only for the burden brought by tinnitus, but also for stress, anxiety and other parameters if there was no indication of an accompanying psychological disorder (Zirke et al., 2013).

A diagnosis of anxiety or psychosomatic disturbances seems to have a particularly negative effect on the degree to which speech recognition improves from the pre- to postoperative stages of CI. The effects of therapy are modulated by mechanisms of neuronal plasticity, as described above, but also by behavioral patterns and dysfunctional coping strategies. Anxiety promotes avoidance behavior and a tendency to withdraw, which can result in a failure to engage in essential types of communication. The trends seen in our psychosomatic observations suggest that a focus on models and therapies related to the physical causes of these conditions places a stronger emphasis on external factors in accounting for the benefits of therapy, while making patients less aware of and less likely to draw on their resources to influence the outcome. For instance, patients should be psychologically encouraged and supported in their use of CI. We assumed that some CI recipients are simply highly focused on the degree of their disability before the implantation, whether or not they are affected by a psychological disturbance (Olze et al., 2011). As their hearing improves, psychological and other types of burdens, which may have been masked by auditory and communication problems, move into the foreground.

### Study limitations

The major limitations of this study are small size and lack of homogeneity in our sample. Of 47 patients, 36 were bilaterally deaf. The remaining 11 patients had a significant hearing loss on the non-CI ear that could not be classified as deafness. This might have affected some of our results, for instance it could have lowered the average scores of anxiety post-implantation. In addition, there may be differences between the bilaterally- and single sided deafened patients with significant hearing loss on non-CI ear, in terms of CIDI psychological diagnoses. Future focus on homogenous groups and increasing the sample number will clarify this issue. The next limitation of our study is the heterogeneity of our sample regarding the duration of hearing loss. The last limitation is the known hypersensitivity of disease detection by CIDI, when compared to classical diagnostic process (Becker et al., 2006; Terber et al., 2010). Despite this fact, this is the first work when CIDI is used to diagnose the patients undergoing cochlear implantation.

### Clinical significance

Since deaf patients have been shown to suffer from a higher psychological stress than the normal-hearing people, psychometric diagnosis and a patient history extended by psychosomatic aspects are indispensable when planning CI. In the presence of manifest psychological disorder (depression, anxiety, somatization disorder) appropriate therapeutic co-operation should be initiated even before the implantation. Apart from establishing the psychological status of a patient before the CI, psychosomatic monitoring should also be carried out after the implantation because of possible occurrence of psychological disorders after the CI, which could make the auditory rehabilitation process more difficult. Over the entire course of treatment, close cooperation of the ORL, head and neck surgeons and audiologists with therapists specialized in psychosomatic medicine is indispensable in order to achieve an optimal result of cochlear rehabilitation.

## Summary

In general, patients suffering from deafness have higher levels of psychological burden than the population as a whole. The standardized tools of psychiatric diagnosis reveal that they are particularly subject to affective disorders and anxiety, as well as psychosomatic illnesses, which are strongly associated with tinnitus. Conditions diagnosed before the surgery do not directly change through the implantation—this can only occur through targeted therapies. According to the sample description (age, sex, and other social variables) none of these factors significantly correlated with the significant improvement in all aspects of hearing abilities measured during this study. However, duration of deafness correlated significantly with the hearing quality (measured by Ol) pre- and post-CI. In addition, CI did have positive influence extending beyond hearing, particularly in many psychosocial aspects of their lives, including self-evaluations of their levels of depression and anxiety, tinnitus, quality of life, and coping strategies, as measured through the questionnaires.

Our results align with current models of the mechanisms that underlie neuronal plasticity, which at least partially account for post-CI improvements in the auditory processing of patients, including reductions in the symptoms of tinnitus, independent of a person's degree of psychological burden. While here the influence of concurrent psychological illnesses and various aspects of a patient's quality of life seem to have a rather indirect effect, it will be crucial to collect further data, such as the degree of hearing loss in the contralateral ear, to capture a better picture of how these factors, which vary highly between individuals, should be integrated into predictions about the potential effectiveness of therapies. This strongly suggests that through the entire process of cochlear implantation, from the original diagnosis through rehabilitation and follow-up care, there is a particular need to provide comprehensive psychosomatic care for patients, especially considering the negative effects that anxiety and other psychosomatic conditions have on the ultimate value of the therapy in their lives.

## Author contributions

PB: study design; analysis and interpretation of data; drafting of manuscript. AS: analysis and interpretation of data; drafting of manuscript. KK: data acquisition; analysis and interpretation of data. SG: data acquisition; data analysis. BM: critical revision; HO: study conception and design; analysis and interpretation of data.

### Conflict of interest statement

The authors declare that the research was conducted in the absence of any commercial or financial relationships that could be construed as a potential conflict of interest. The reviewer ND and handling Editor declared their shared affiliation, and the handling Editor states that the process nevertheless met the standards of a fair and objective review.
